# Sirt1 overexpression attenuates Western‐style diet‐induced aortic stiffening in mice

**DOI:** 10.14814/phy2.15284

**Published:** 2022-05-13

**Authors:** Venkateswara R. Gogulamudi, Daniel R. Machin, Grant D. Henson, Jisok Lim, Richard C. Bramwell, Jessica R. Durrant, Anthony J. Donato, Lisa A. Lesniewski

**Affiliations:** ^1^ Department of Internal Medicine University of Utah Salt Lake City Utah USA; ^2^ 7823 Department of Nutrition and Integrative Physiology Florida State University Tallahassee Florida USA; ^3^ Dallas Tissue Research Dallas Texas USA; ^4^ Geriatrics Research Education and Clinical Center Veteran’s Affairs Medical Center Salt Lake City Utah USA; ^5^ Department of Nutrition and Integrative Physiology University of Utah Salt Lake City Utah USA

**Keywords:** aorta, arterial stiffness, Sirt1, sirtuins, Western diet

## Abstract

Increased arterial stiffness is a cardiovascular disease risk factor in the setting of advancing age and Western diet (WD) induced obesity. Increases in large artery stiffness, as measured by pulse wave velocity (PWV), occur within 8 weeks of WD feeding in mice. Sirtuin‐1 (Sirt1), a NAD‐dependent deacetylase, regulates cellular metabolic activity and activation of this protein has been associated with vasoprotection in aged mice. The aim of the study was to elucidate the effect of global Sirt1 overexpression (Sirt^tg^) on WD‐induced arterial stiffening. Sirt1 overexpression did not influence PWV in normal chow (NC) fed mice. However, PWV was higher in wild‐type (WT) mice (*p* < 0.04), but not in Sirt^tg^ mice, after 12 weeks of WD and this effect was independent of changes in blood pressure or the passive pressure diameter relation in the carotid artery. Overexpression of Sirt1 was associated with lower collagen and higher elastin mRNA expression in the aorta of WD fed mice (both *p* < 0.05). Although MMP2 and MMP3 mRNA were both upregulated in WT mice after WD (both *p* < 0.05), this effect was reversed in Sirt^tg^ mice compared to WT mice fed WD (both *p* < 0.05). Surprisingly, histologically assessed collagen and elastin quality were unchanged in the aortas of WT or Sirt^tg^ mice after WD. However, Sirt^tg^ mice were protected from WD‐induced glucose intolerance, although there was no difference in insulin tolerance between groups. These findings demonstrate a vasoprotective effect of Sirt1 overexpression that limits the increase in arterial stiffness in response to consumption of a WD.

## Graphical abstract

Sirt1 attenuated Western diet‐induced aortic stiffness.

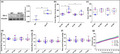

## 1 INTRODUCTION

Western diet and obesity are associated with cardiovascular disease (CVD), the leading cause of morbidity and mortality worldwide (Benjamin et al., 2019). Arterial dysfunction, characterized by endothelial dysfunction and increased large artery stiffness, occurs with consumption of WD feeding (Benjamin et al., 2019; Donato et al., 2012; Weisbrod et al., 2013) and is an independent risk factor for CVD (Lacy et al., 2004; Scuteri et al., 2005). Indeed, long‐term ingestion of WD leads to increases in large artery stiffness resulting in increased pulse wave velocity (PWV) (Henson et al., 2014; Santana et al., 2014) and hypertension (Weisbrod et al., 2013). These outcomes are associated with morphological changes in the vasculature such as alterations in vessel diameter and wall thickness (Dantas et al., 2014) and/or changes in structural components of the arterial wall, such as collagen and elastin (Henson et al., 2014). With the high prevalence of excessive dietary fat consumption (Santana et al., 2014) and obesity (Wildman et al., 2003), it is essential to understand the mechanisms underlying increases in arterial stiffness and to develop strategies to combat increased CVD risk in these populations.

Sirtuin‐1 (Sirt1) belongs to the NAD^+^ ‐dependent class III histone deacetylases (Tajbakhsh & Sokoya, 2012). In mammals, seven sirtuin homologs designated as Sirt1‐Sirt7 have been identified, all seven sirtuins share a common catalytic core (Lu et al., 2018). Sirtuins have been implicated in the regulation of various biological systems in association with caloric restriction, metabolism, aging, cancer, cell differentiation, chromosomal stability, stress resistance, inflammation, DNA repair, tissue fibrosis, mitochondrial biogenesis, and apoptosis (Brochier et al., 2013; Lee & Yang, 2017; Michan & Sinclair, 2007; Ozawa et al., 2010; Wojcik et al., 2009; Zhang et al., 2017). Sirt1 plays a regulatory role through deacetylation of non‐histone proteins such as endothelial nitric oxide (eNOS), Forkhead Box O (FOXO), liver x receptor (LXR), and tumor protein p53 (P53) and peroxisome proliferator‐activated receptor‐gamma coactivator alpha (PGC1 alpha) (Brunet et al., 2004; Chen, Su, et al., 2020; Li et al., 2007; Mattagajasingh et al., 2007; Rodgers et al., 2005; Vaziri et al., 2001). Importantly, recent studies have also demonstrated a detrimental effect of reduced Sirt1 on endothelial function that was associated with a reduced activation of eNOS and impaired NO production in aged mice (Donato et al., 2011; Gogulamudi et al., 2018). In contrast, pharmacological activation of Sirt1 has been shown to reverse endothelial dysfunction that was associated with a reduction of both ROS production and inflammation in aged mice (Gano et al., 2014). In addition, NO produced by eNOS also promotes smooth muscle relaxation and inhibits adverse arterial remodeling (Csiszar et al., 2002). Sirt1 overexpression in vascular smooth muscle cells represses neointima formation in response to vascular injury (Li et al., 2011). Similarly, smooth muscle cell Sirt1 overexpression prevents angiotensin II (Ang II)‐induced activation of matrix metalloproteinase (MMP) and neointimal remodeling (Wan et al., 2014). Moreover, treatment with resveratrol, an indirect Sirt1 activator, downregulates Ang II type 1 receptor expression in VSMC through Sirt1 activation both in vivo and in vitro (Miyazaki et al., 2008; Zurlo et al., 2018). Previous studies have also demonstrated VSMC and whole body Sirt1 overexpression protects against long term (8 months) Western (high‐fat high‐sucrose [HFHS]) diet induced aortic stiffening (Fry et al., 2016), however, the effects of Sirt1 overexpression on arterial stiffness and vascular morphology in the setting of HFHS diet is not known.

In the present study, we tested the hypothesis that Sirt1 overexpression will attenuate WD induced aortic stiffening. To do so, we assessed in‐vivo aortic stiffness by PWV in a global Sirt1 overexpressing transgenic mouse model after the consumption of either NC or WD for 12 weeks as previously described (Favero et al., 2020; Henson et al., 2014). In addition, we examined the impact of Sirt1 overexpression on changes in blood pressure as well as examined the impact of Sirt1 overexpression on WD associated alterations in the key arterial structural proteins, collagen and elastin, and vascular morphology.

## 2 MATERIAL AND METHODS

### 2.1 Animals

Wild‐type (WT) C57BL/6 mice were obtained from Charles River Laboratories (Wilmington, MA) and whole‐body Sirt1 transgenic overexpressing (Sirt^tg^) mice on a C57BL/6 background were provided by Dr. Gu (Columbia University, New York) (Banks et al., 2008). WT mice were mated with Sirt^tg^ mice to generate littermates of both genotypes at the animal facility of VA Medical Center (VAMC‐SLC), Salt Lake City. All the mice were housed at the animal facility of VAMC‐SLC on a 12 h light/dark cycle and fed water and food ad libitum. Six to eight month old male and female mice weighing approximately 25–27 g were used in this study. Mice were fed a normal chow (NC) (16% kcal from fat, 55% carbohydrate, 29% protein, 0% sucrose normal chow #TD.8640 Harlan Teklad 22/5 Standard Rodent Chow) or a commercially available high fat high sucrose (HFHS) chow (41% kcal from saturated/total fat, 41% from carbohydrate (including 17% sucrose), 18% protein, Harlan Teklad Adjusted Fat diet #TD.96132) ad libitum for 12 weeks prior to sacrifice (Donato et al., 2011; Henson et al., 2014). Hereafter we are mentioning our HFHS diet as WD. When possible, outcomes were assessed at NC and WD.

### 2.2 Western blotting

Thoracic aortas from NC and WD fed WT and Sirt^tg^ mice were dissected, cleared of the perivascular adipose and stored in liquid nitrogen. Aortic lysates were prepared in RIPA buffer by physical disruption with a homogenizer and supernatants were collected. After the total protein quantification, equal amounts of protein (20 µg) were loaded in 4%–12%—Criterion XT Bis‐Tris protein gels (Bio‐Rad, CA) and transferred to nitrocellulose membranes. The membranes were blocked with 1% BSA with TBST before incubation with Sirt1 primary antibody (1:200, Santa Cruz, CA) at 4°C overnight. Membranes were then washed three times with TBST and incubated with secondary antibody for 1 h at room temperature. Blots were developed by super signal ECL reagent and bands were visualized using a gel documentation system. To normalize the protein loading differences, vinculin protein was used as housekeeping protein expression and data was normalized to the mean of the WT group.

### 2.3 Aortic pulse wave velocity (PWV)

Aortic PWV was measured before and 12 weeks after initiation of WD as described previously (Donato et al., 2013; Henson et al., 2014). Briefly, mice were anesthetized with 2% isoflurane in a closed compartment anesthesia machine (V3000PK, Parkland Scientific) for ~1–3 min. Throughout the procedure anesthesia was maintained via a nose cone and mice were secured in a supine position on a heated board (~35°C) to maintain body temperature. PWV was measured with 20‐MHz Doppler probes (Indus Instruments) at the transverse aortic arch and ~4 cm distal at the abdominal aorta and collected using WinDAQ Pro+software (DataQ Instruments). Absolute pulse arrival times were indicated by the sharp upstroke, or foot, of each velocity waveform analyzed with WinDAQ Waveform Browser (DataQ Instruments) and distance between the probes was measured using a scientific caliper. Velocities were then calculated as the quotient of the separation distance (~3 cm) and the difference in absolute arrival times.

### 2.4 Blood pressure

Blood pressure was measured in NC and WD fed WT and Sirt^tg^ mice via non‐invasive tail‐cuff (CODA System, Kent Scientific) technique as described previously (Donato et al., 2013). Briefly, mice were acclimated to the restrainers and then placed on a heating unit to reach a steady body (skin and tail) temperature (37°C) in a quiet room. Systolic blood pressure (SBP), diastolic blood pressure (DBP), and mean arterial pressure (MAP) were calculated from the average of 15 recordings.

### 2.5 Passive pressure diameter relations

To evaluate the passive properties of the large arteries, carotid arteries were excised, cleared of surrounding tissues and cannulated onto glass micropipettes in the stage of a pressure myograph system containing calcium‐free physiological salt solution. Luminal diameter and wall thickness were recorded at 68 mmHg intraluminal pressure. The passive pressure‐diameter relation was then measuring the luminal diameter of the artery when subjected to a range of intraluminal pressures from 5 to 100 mmHg as previously described (Hazra et al., 2019).

### 2.6 Quantitative real‐time PCR

Relative mRNA levels were measured by real‐time reverse‐transcriptase polymerase chain reaction from the aortic tissues. RNA was isolated using Qiazole (Qiagen) method as per the manufacturer's instructions. Total RNAs were converted to cDNA using the QuantiTect cDNA synthesis kit (Qiagen). Real‐time qPCR was performed using EvaGreen RT–qPCR kit (Bio‐Rad). Relative mRNA expression was quantified with the comparative cycle threshold (Ct) method and expressed as 2‐ΔΔCt. The sequences of primers were summarized in Table 1.

**TABLE 1:** Forward and reverse sequence of primers used to amplify specific mRNA in the aortic tissue of WT and Sirt^tg^ mice

Target mRNA	Forward sequence	Reverse sequence
MMP2	5’‐CAAGTTCCCCGGCGATGTC−3’	5’‐CAAGTTCCCCGGCGATGTC−3’
MMP3	5’‐ACATGGAGACTTTGTCCCTTTTG−3’	5’‐TTGGCTGAGTGGTAGAGTCCC−3’
Elastin	5’‐TCCATCCGCCCTGGTGTAT−3’	5’‐TGGCAGTCTGGTCCTCTAAAG−3’
Collagen	5’‐CGATGGATTCCCGTTCGAGT−3’	5’‐CGATCTCGTTGGATCCCTGG−3’
18s	5’‐TAGAGGGACAAGTGGCGTTC−3’	5’‐CGCTGAGCCAGTCAGTGT−3’

### 2.7 Vessel morphology

Mice were euthanized by exsanguination via cardiac puncture while maintained under isoflurane anesthesia. Thoracic aortas were excised and placed in physiological saline at 4°C. With the aid of a dissecting microscope, perivascular tissues were cleared and ~2 millimeter (mM) rings were cut from the descending thoracic aorta distal to the greater curvature. Each aortic sample was bisected and embedded in the transverse plane to create two tissue sections per slide. Three slides per block were sectioned (4 μm) and stained with hematoxylin and eosin (H&E), Verhoeff Van Gieson (VVG), or Movat's pentachrome (MP) stain. All slides were evaluated by an ACVP‐board certified veterinary pathologist in a blinded fashion. Glass slides were imaged using an EasyScan Pro 6 digital slide scanner to create whole slide images, which were imported into QuPath software (Bankhead et al., 2017) for measurements. Aortic wall measurements were performed on H&E‐stained slides. The mean area was determined by annotating the luminal and external borders of the aortic wall (endothelium and external elastic lamina) and measuring the area of each section (μm^2^); these two measurements were averaged to obtain the mean for that sample. Mean diameter (μm) of each aortic section was determined by averaging the long and short axis measurements (from external elastic lamina to contralateral external elastic lamina) for each section. Medial (wall) thickness (μm) was measured by determining the distance between the internal elastic lamina and external elastic lamina in five sites per section (10/animal); all sites were averaged to obtain a mean medial thickness. In addition, the ratio of aortic medial thickness to aortic diameter was also assessed as an overall indicator of arterial remodeling. The number of smooth muscle nuclei were counted in each section as well. The ratio of smooth muscle nuclei to the medial area was determined. As histologically prepared arteries will not have the same morphometric measurements as an unfixed artery at physiological pressures, we are reporting morphometric measurements as ratios whenever possible.

Changes to the composition of the aorta were assessed with MP‐ and VVG‐stained slides. Using MP‐stained slides, in which smooth muscle stains red‐purple, collagen stains yellow, and proteoglycan stains aqua blue, alterations in the collagen and proteoglycan content of tunica media were assessed by determining the area of the tunica media staining for these extracellular matrix components. This was scored by an ACVP‐board certified veterinary pathologist in a blinded fashion on a scale of 0–3, with 1 = <10% area affected, 2 = 10%–50% area affected, 3 = >50% area affected. Two sections of aorta were assessed from each animal and the average score was used in the analysis. Using VVG stained slides, elastin breaks, defined as any discontinuity in the elastic laminae, were counted in each aortic section and averaged per animal. Elastin breaks were also expressed normalized to the aortic medial area. Elastin loss was defined as an area in which absent elastic laminae were expected to be present and scored on a scale of 0 to 5, with 0 = no foci of loss although breaks may be present, 1 = <10% loss, 2 = 10%–25% loss, 3 = 26%–50% loss, 4 = 51%–75% loss, and 5 = >75% loss. Areas within or immediately adjacent to blood vessel branch points were not considered for measurement/counts in these analyses as alterations in wall thickness and elastin or extracellular content are seen in these zones.

### 2.8 Metabolic assessment

Glucose tolerance tests (GTTs) were performed in fasted (6 h) mice after an intraperitoneal injection of D‐glucose (2 g/kg of body weight) as described previously (Hazra et al., 2019; Trott et al., 2021). Insulin tolerance tests (ITTs) were performed in fasted (6 h) mice after an intraperitoneal injection of insulin at a dose of 1.0 U/kg of body weight as described (Frost & Olson, 2011). Blood samples were collected at time 0, 15, 30, 60, 90, and 120 min after injections via tail nick. Glucose was measured using a Precision Xceed Pro Glucose analyzer (Abbott Laboratories).

### 2.9 Statistics

To determine if differences existed between genotypes and diets, two‐way ANOVAs or two‐way repeated‐measures ANOVAs were performed where appropriate. Bonferroni's multiple comparison post hoc tests were performed for planned comparisons. Kruskal–Wallis One Way Analysis of Variance on Ranks was used to determine differences between groups for scaled variables. An unpaired *t*‐test was conducted to determine the difference in Sirt1 protein levels in the thoracic aorta between the WT and Sirt^tg^. All the data are expressed as mean ± SEM unless otherwise noted, significance was set as *p* < 0.05.

## 3 RESULTS

### 3.1 Animal characteristics

**FIGURE 1:**
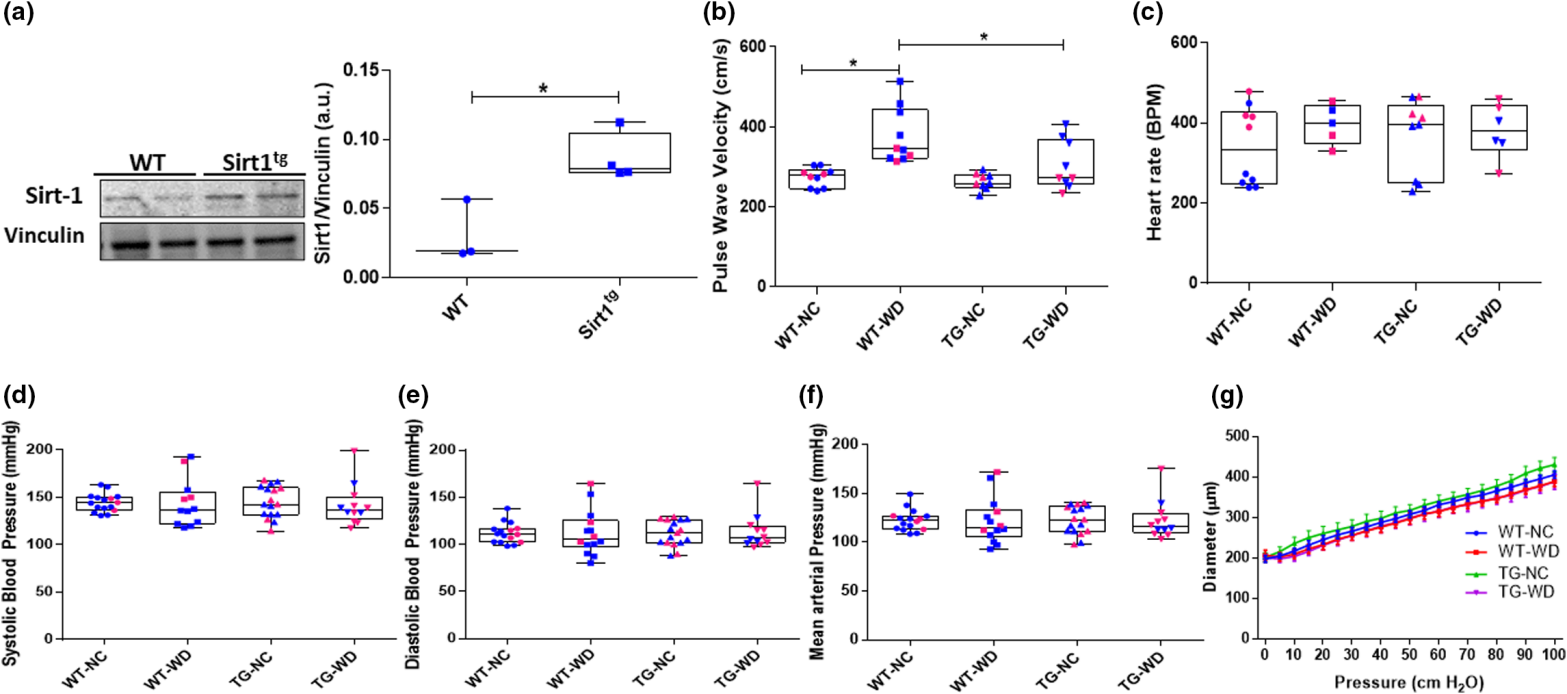
(a) Sirt1 protein expression measured by western blotting, in thoracic aorta excised from WT and Sirt1^tg^ mice (b) Aortic pulse wave velocity (PWV), (c) heart rate, (d) systolic blood pressure (SBP), (e) diastolic blood pressure (DBP), (f) mean arterial pressure (MAP), and the (g) passive pressure diameter relation in excised carotid arteries of WT and Sirt^tg^ littermates fed normal chow (NC) or 12 weeks of Western diet (WD). Individual data points are separated by sex are indicated in blue (male) and pink (female). *Denotes a significant difference within genotype, *p* < 0.05. Values are mean ± SEM

There was a significant difference in aortic Sirt1 protein expression, Sirt1 protein expression in the thoracic aorta was higher in NC fed Sirt^tg^ compared to WT mice (*p* < 0.01, Figure 1a). Body mass was also different between groups (genotype: *p* = 0.004, diet: *p* = 0.907, interaction: *p* = 0.592) with mass higher after WD in both WT (*p* = 0.016) and Sirt^tg^ (*p* = 0.035) mice compared to NC fed mice. Likewise, tissue mass of the heart (genotype: *p* = 0.765, diet: *p* = 0.663, interaction: *p* = 0.507), liver (genotype: *p* = 0.074, diet: *p* = 0.583, interaction: *p* = 0.395), spleen (genotype: *p* = 0.603, diet: *p* = 0.107, interaction: *p* = 0.012), kidney (genotype: *p* = 0.038, diet: *p* = 0.749, interaction: *p* = 0.941), WAT (genotype: *p* < 0.001, diet: *p* = 0.320, interaction: *p* = 0.432) and lung (genotype: *p* = 0.055, diet: *p* = 0.388, interaction: *p* = 0.985) all differed between groups. There was no effect of WD on heart (*p* = 0.765), liver (*p* = 0.074), spleen (*p* = 0.972) and lung (*p* = 0.055) mass in Sirt^tg^ mice. Although WT mice had higher heart and liver mass (*p* < 0.05) after WD diet compared to NC fed WT mice. In contrast, compared to NC fed mice, the mass of the kidney (*p* < 0.038) and WAT (*p* < 0.001) was higher in both WT and Sirt^tg^ WD fed mice (Table 2).

**TABLE 2:** Body, heart, liver, spleen, lung, kidney, and epididymal white adipose tissue (WAT) mass in WT and Sirt^tg^ normal chow (NC) and Western diet (WD)‐fed mice

	WT – NC	WT ‐ WD	Sirt^tg^ ‐ NC	Sirt^tg^ ‐ WD
Male: female *N*	12:7	10:11	13:9	11:10
Body mass (g)	25.79 ± 1.498	35.73 ± 3.060*	26.91 ± 1.490	33.98 ± 2.492*
Heart mass (g)	0.120 ± 0.020	0.137 ± 0.008*	0.140 ± 0.029	0.133 ± 0.016
Liver mass (g)	1.270 ± 0.087	1.800 ± 0.221*	1.330 ± 0.033	1.525 ± 0.214
Spleen mass (g)	0.070 ± 0.012	0.125 ± 0.013	0.130 ± 0.200	0.100 ± 0.012
Lung mass (g)	0.210 ± 0.040	0.162 ± 0.008	0.230 ± 0.033	0.183 ± 0.010
Kidney mass (g)	0.230 ± 0.048	0.337 ± 0.057*	0.210 ± 0.018	0.325 ± 0.042*
WAT mass (g)	0.420 ± 0.112	1.344 ± 0.285*	0.470 ± 0.099	1.758 ± 0.182*

Note

Values are mean ± SEM.

* *p* < 0.05.

### 3.2 Aortic stiffness, blood pressure, and passive pressure diameter relation

There was a significant difference in aortic stiffness, assessed by PWV (genotype: *p* < 0.001, diet: *p* = 0.014, interaction: *p* = 0.067). Sirt1 overexpression did not influence aortic stiffness in NC fed mice (*p* = 0.999). However, WD increased aortic stiffness in WT mice (*p* = 0.003), but this effect was attenuated in the Sirt^tg^ mice (*p* = 0.491) (Figure 1b). Anesthetized heart rate measured during PWV assessments was similar between groups (Figure 1c, genotype: *p* = 0.274, diet: *p* = 0.917, interaction: *p* = 0.556). Likewise, there were no significant differences between the groups for SBP (Figure 1d, genotype: *p* = 0.742, diet: *p* = 0.897, interaction: *p* = 0.787), DBP (Figure 1e, genotype: *p* = 0.777, diet: *p* = 0.930, interaction: *p* = 0.832) or MAP (Figure 1f, genotype: *p* = 0.993, diet: *p* = 0.975, interaction: *p* = 0.909). To determine if changes in the passive mechanical properties of the large arteries could underlie alterations in stiffness observed in vivo, we performed passive pressure‐diameter responses on isolated cannulated carotid arteries. However, we found no differences in the pressure diameter relation between groups (group, pressure and interaction: *p* > 0.999) (Figure 1g).

### 3.3 Aortic collagen, elastin, MMP2, and MMP3 mRNA expression

**FIGURE 2:**
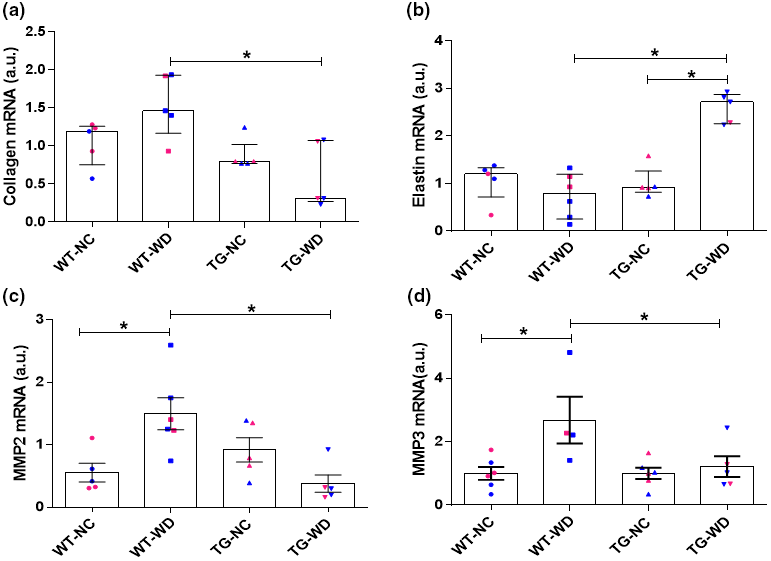
(a) Collagen, (b) elastin, (c) MMP2, and  (d) MMP3 mRNA expression in thoracic aorta excised from WT and Sirt^tg^ mice fed either normal chow (NC) or Western diet (WD) for 12 weeks. Individual data points separated by sex are indicated in blue (male) and pink (female). *Denotes a significant difference, *p* < 0.05. Values are mean ± SEM

To explore potential mechanisms underlying the observed alterations in aortic stiffness, we assessed gene expression for the aortic structural components (collagen and elastin) and extracellular matrix enzymes, MMP2 and MMP3. There was a significant main effect for genotype in collagen mRNA expression (Figure 2a, genotype: *p* = 0.506, diet: *p* = 0.002, interaction: *p* = 0.026). Although collagen mRNA did not differ between genotypes in NC fed mice (*p* = 0.999) or after WD in either the WT (*p* = 0.253) or Sirt^tg^ (*p* = 0.999) mice, expression was lower between WT and Sirt^tg^ mice after WD (Figure 2a, *p* = 0.004). Likewise, there was a significant group difference in elastin expression (Figure 2b, genotype: *p* = 0.002, diet: *p* < 0.001, interaction: *p* < 0.001), with Sirt^tg^ mice demonstrating increased elastin mRNA expression compared to both NC fed Sirt^tg^ and WD fed WT (*p* < 0.001) after WD. WD was without effect in WT (*p* > 0.999) mice (Figure 2b). There was a significant group effect for MMP2 and MMP3 expression (*p* = 0.001 and *p* = 0.043). WD led to an increase in MMP2 (genotype: *p* = 0.333, diet: *p* = 0.078, interaction: *p* = 0.001) and MMP3 (genotype: *p* = 0.027, diet: *p* = 0.008, interaction: *p* = 0.043) in WT mice (Figure 2c–d) that was ameliorated in Sirt^tg^ mice (Sirt^tg^ NC vs. WT, *p* = 0.477 and *p* > 0.999) (Figure 2c–d).

### 3.4 Arterial structural proteins

**FIGURE 3:**
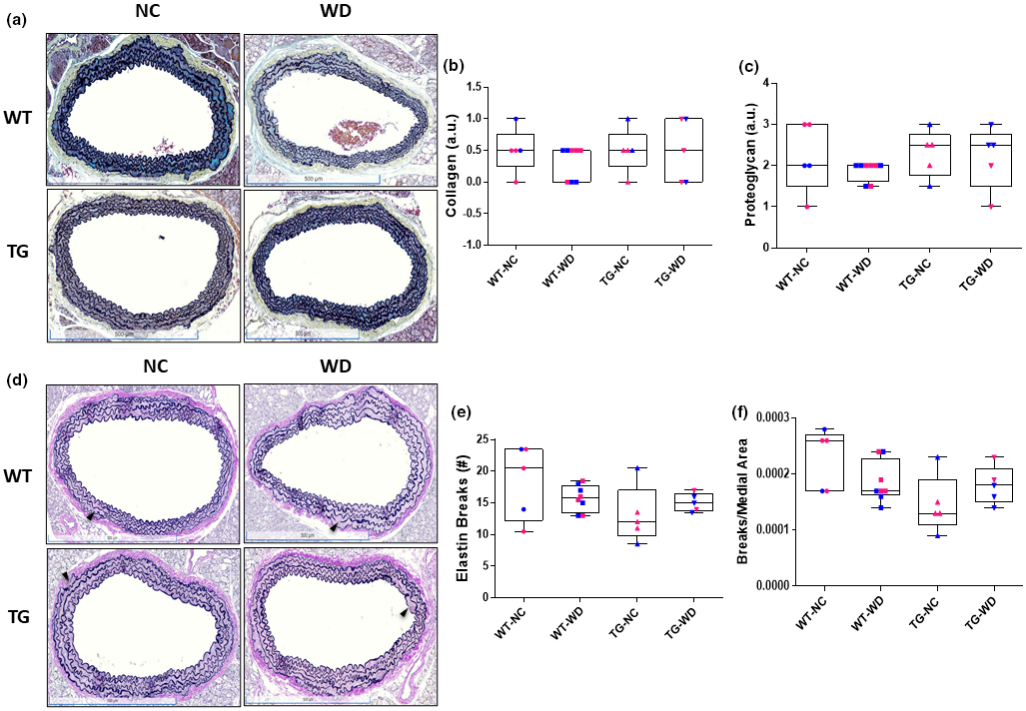
(a) Representative images of aortic sections stained with Movat's pentachrome (MP). (b) Collagen content and (c) proteoglycan content, assessed in MP stained sections of thoracic aorta excised from WT and Sirt^tg^ mice fed either normal chow (NC) or Western diet (WD) for 12 weeks. (d) Representative images of aortic sections stained with Verhoeff's Van Gieson (VVG). (e) Elastin breaks (arrowheads) and (f) ratio of elastin breaks in aortic medial area to aortic area in VVG stained aortic sections excised from WT and Sirt^tg^ mice fed either normal chow (NC) or Western diet (WD) for 12 weeks. Individual data points are separated by sex are indicated in blue (male) and pink (female)

To assess the impact of Sirt1 overexpression on WD‐associated alterations in arterial structural components, we evaluated collagen and proteoglycan within the tunica media in MP stained aortic sections as well as aortic elastin quality by VVG staining (Figure 3a). There were no differences in scoring for collagen (Figure 3b, genotype: *p* = 0.548, diet: *p* = 0.548, interaction: *p* = 0.548) or proteoglycan (Figure 3c, genotype: *p* = 0.413, diet: *p* = 0.413, interaction: *p* = 0.663) within the tunica media.

We next assessed the impact of Sirt1 overexpression on WD‐associated alterations in arterial elastin quality using VVG stained sections of aorta. Elastin loss, defined as loss of elastin in an area that it would be expected, was not different (genotype: *p* > 0.999, diet: *p* > 0.999, interaction: *p* > 0.999), with all groups demonstrating <10% of expected elastin area lost. The number of elastin breaks was also assessed and is represented as both an absolute number and as a ratio to the medial area. Likewise, there were no differences for total number of breaks (Figure 3e, genotype: *p* = 0.838, diet: *p* = 0.073, interaction: *p* = 0.154). However, the ratio of breaks to medial area was influenced by the diet (Figure 3f, genotype: *p* = 0.811, diet: *p* = 0.030, interaction: *p* = 0.052).

### 3.5 Aortic morphology

**FIGURE 4:**
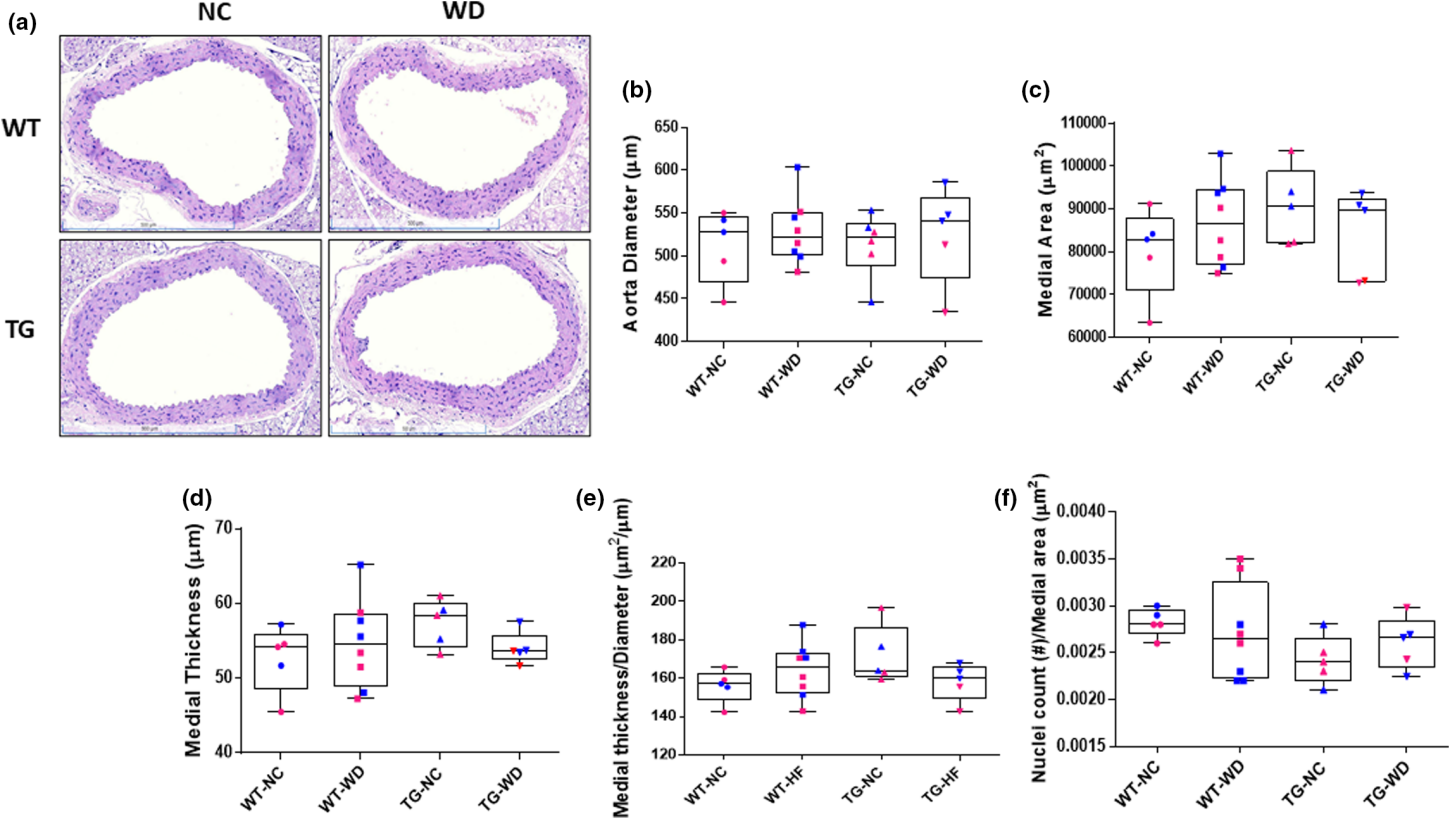
(a) Representative images of aortic sections stained with hematoxylin and eosin (H&E). (b) Aortic diameter, (c) medial area, (d) medial thickness, (e) ratio of medial thickness to aortic diameter, and (f) ratio of nuclei count to medial area in H&E stained thoracic aorta sections excised from WT and Sirt^tg^ mice fed either normal chow (NC) or Western diet (WD) for 12 weeks. Individual data points are separated by sex are indicated in blue (male) and pink (female)

To gain initial insight into the impact of Sirt1 overexpression on alterations in aortic morphology (Figure 4a) we assessed aortic diameter, medial (wall) area and medial thickness as well as the ratio of the medial area to aortic diameter. We also assessed the total number of nuclei within the tunica media in absolute terms and relative to wall area as a gross assessment of smooth muscle hyperplasia versus hypertrophy. Neither the aortic diameter (Figure 4b, genotype: *p* = 0.671, diet: *p* = 0.772, interaction: *p* = 0.600), medial area (Figure 4c, genotype: *p* = 0.962, diet: *p* = 0.376, interaction: *p* = 0.140), medial thickness (Figure 4d, genotype: *p* = 0.745, diet: *p* = 0.297, interaction: *p* = 0.176), nor the medial thickness to aortic diameter ratio (Figure 4e, genotype: *p* = 0.613, diet: *p* = 0.375, interaction: *p* = 0.056) differed. Likewise, there was no group difference in the smooth muscle nuclei count within the tunica media when expressed either in absolute terms (genotype: *p* = 0.764, diet: *p* = 0.413, interaction: *p* = 0.777) or in relation to the aortic diameter (Figure 4f, genotype: *p* = 0.800, diet: *p* = 0.116, interaction: *p* = 0.351). These data do not suggest that gross changes in medial wall morphology underlie the protection against WD‐associated aortic stiffening that was afforded by Sirt‐1 overexpression.

### 3.6 Sirt1 overexpression protected against WD‐induced glucose intolerance

**FIGURE 5:**
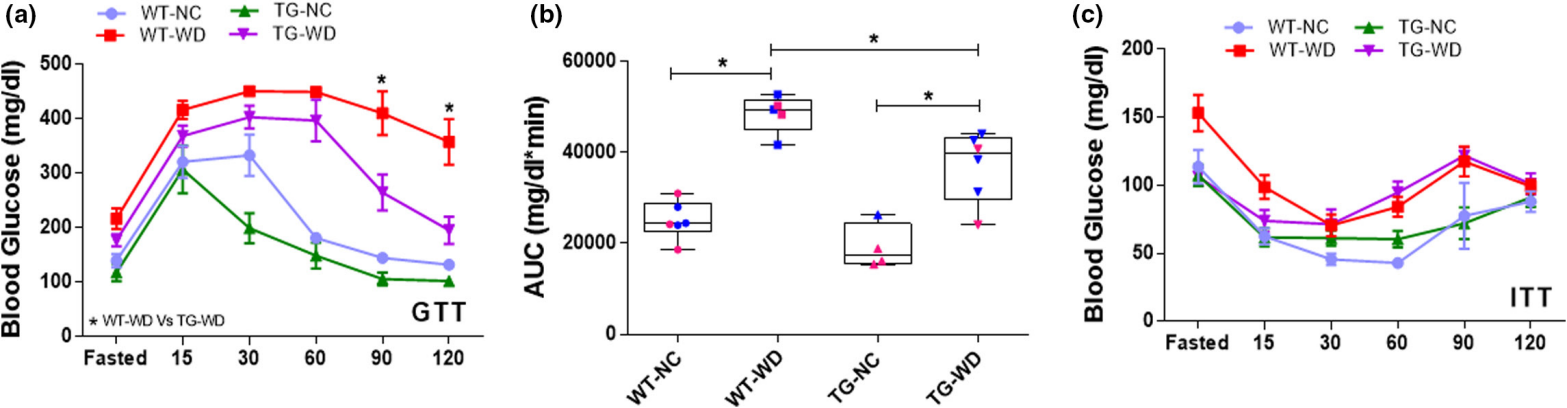
(a) Blood glucose during a glucose tolerance test (GTT) (2 g/kg, ip), and (b) area under curve (AUC) during the GTT (c) insulin tolerance test (ITT) (1 U/kg, ip). Individual data points are separated by sex are indicated in blue (male) and pink (female). *Denotes a significant difference, *p* < 0.05. Values are mean ± SEM

To examine metabolic function, we performed glucose‐ and insulin‐ tolerance tests in NC‐ and WD‐fed WT and Sirt^tg^ mice. Time response curves during the glucose tolerance test differed between groups (group, diet, and interaction: *p* < 0.0001). Twelve weeks of WD resulted in glucose intolerance in both WT (*p* < 0.0001 NC versus WD in WT) and Sirt^tg^ (*p* < 0.0001 NC versus WD in Sirt^tg^) mice as indicated by elevated glucose during the GTT. However, WD fed Sirt^tg^ mice demonstrated lower glucose during the GTT compared to WD fed WT mice (*p* > 0.999) and these differences occurred primarily at the later time points such as 90 min (*p* < 0.010) and 120 min (*p* < 0.005), suggesting that the WD fed Sirt^tg^ mice have a better ability to clear the elevated blood glucose compared to WT control (Figure 5a). When expressed as area under the curve (AUC), we also found a significant group difference (group: *p* = 0.001, diet: *p* = 0.210, interaction: *p* = 0.636). While there was no difference between NC fed WT and Sirt^tg^ mice (*p* = 0.999), WD elevated the AUC in both WT and Sirt^tg^ mice (*p* = 0.006 and *p* = 0.015). However, WD fed Sirt^tg^ mice demonstrated a lower AUC compared to WD fed WT mice (*p* = 0.028). Taken together, this data suggests that the attenuation in arterial stiffness is concomitant with an improvement in glucose tolerance in WD fed Sirt^tg^ mice (Figure 5b). Similar to before the GTT, fasted blood glucose differed between groups (group: *p* = 0.0001, diet: *p* < 0.0001 interaction: *p* = 0.314) and tended to be higher in WT mice after WD compared to all other groups (all *p *≥ 0.0001), however, there were no differences in the time response curves during the insulin tolerance test (group, time and interaction: *p* = 0.171, Figure 5c).

### 3.7 Sex differences

Table 3 provides means, standard errors, and *p* values two‐way ANOVAs for primary outcomes separated by sex. PWV was the only variable that demonstrated different outcomes based on sex. We found that in both male (*p* = 0.0014) and female (*p* = 0.026) mice PWV was higher after WD in WT mice, but that PWV was lower in Sirt^tg^ compared to WT mice after WD only in the female (*p* = 0.003) mice.

**TABLE 3:** Means and standard errors of primary outcomes separated by sex

		WT		Sirt^tg^		*p*‐value		
		NC	WD	NC	WD	Genotype	Diet	Interaction
PWV (cm/s)	Male (*N* = 7,7,6,6)	272.0 ± 10.66	396.2 ± 28.41^b^	259.3 ± 9.30	327.0 ± 26.01	*p* = 0.001	*p* = 0.062	*p* = 0.188
	Female (*N* = 3,3,3,3)	280.1 ± 3.83	329.6 ± 9.47^b^	271.1 ± 7.53	260.0 ± 12.6^a^	*p* = 0.064	*p* = 0.002	*p* = 0.009
HR (bpm)	Male (*N* = 6,2,6,3)	285.0 ± 33.22	415.8 ± 16.26	329.7 ± 40.53	370.7 ± 17.46	*p* = 0.070	*p* = 0.996	*p* = 0.322
	Female (*N* = 4,3,3,3)	425.7 ± 18.85	384.3 ± 36.80	432.8 ± 16.28	391.1 ± 58.83	*p* = 0.2623	*p* = 0.8452	*p* = 0.996
SBP (mmHg)	Male (*N* = 13,9,10,4)	144.4 ± 2.94	138.1 ± 8.01	145.4 ± 4.54	135.7 ± 5.61	*p* = 0.888	*p* = 0.140	*p* = 0.746
	Female (*N* = 3,3,7,8)	144.3 ± 2.49	162.0 ± 13.06	144.6 ± 8.48	151.1 ± 12.86	*p* = 0.649	*p* = 0.308	*p* = 0.635
Aortic diameter (µm)	Male (*N* = 2,4,3,3)	535.1 ± 6.950	538.3 ± 23.99	510.6 ± 32.83	558.5 ± 13.91	*p* = 0.679	*p* = 0.538	*p* = 0.785
	Female (*N* = 3,4,3,2)	496.8 ± 30.11	519.5 ± 14.65	515.5 ± 7.28	474.0 ± 39.50	*p* = 0.687	*p* = 0.687	*p* = 0.192
Medial area (µm^2^)	Male (*N* = 2,4,2,3)	870 ± 414	919 ± 557	923 ± 168	915 ± 118	*p* = 0.638	*p* = 0.689	*p* = 0.581
	Female (*N* = 3,4,3,2)	754 ± 620	817 ± 327	892 ± 718	731 ± 256	*p* = 0.224	*p* = 0.6481	*p* = 0.0741
Nuclei/Medial area (#/µm^2^)	Male (*N* = 2,4,2,3)	0.002 ± 6.667	0.002 ± 0.001	0.002 ± 0.003	0.002 ± 0.001	*p* = 0.235	*p* = 0.398	*p* = 0.120
	Female (*N* = 3,4,3,2)	0.002 ± 6.667	0.003 ± 0.001	0.002 ± 5.774	0.002 ± 0.002	*p* = 0.145	*p* = 0.120	*p* = 0.985
Collagen (a.u.)	Male (*N* = 2,4,2,2)	0.750 ± 0.250	0.300 ± 0.122	0.750 ± 0.250	0.500 ± 0.500	*p* = 0.208	*p* = 0.703	*p* = 0.703
	Female (*N* = 3,4,3,3)	0.333 ± 0.166	0.250 ± 0.144	0.333 ± 0.166	0.500 ± 0.288	*p* = 0.593	*p* = 0.747	*p* = 0.747
Elastin breaks (#)	Male (*N* = 2,4,2,3)	18.75 ± 4.75	16.00 ± 1.17	11.00 ± 2.50	14.83 ± 0.72	*p* = 0.803	*p* = 0.070	*p* = 0.159
	Female (*N* = 2,4,3,2)	18.17 ± 3.93	15.50 ± 1.04	14.50 ± 3.01	15.50 ± 1.50	*p* = 0.765	*p* = 0.516	*p* = 0.516

Note

*p* values represent results of 2‐way ANOVAs with genotype and diet as factors for each sex.

^a^ Difference between genotypes within diet.

^b^ Difference between diet within genotype, *p* < 0.05.

## 4 DISCUSSION

The novel findings of the present study are that Sirt1 overexpression protects against WD‐induced large artery stiffening and that this is associated with a protection against arterial wall expression of structural components, extracellular matrix enzymes, and glucose intolerance. Specifically, we found that Sirt1 overexpression lowers collagen mRNA expression, and prevents an increase in extracellular matrix protein 2, 3 mRNA in response to WD. Likewise, Sirt1 overexpression also protects against WD‐induced glucose intolerance. Interestingly, despite the changes in mRNA observed, this protection occurred independently of changes in collagen or elastin or the morphology of the arterial wall. Our findings suggest that Sirt1 activation may be a viable therapeutic strategy to combat arterial stiffening in the face of obesity/high fat diet consumption.

Previous studies have demonstrated that Sirt1 can play a protective role against experimental hypertension. For example, vascular remodeling in response to angiotensin II‐induced hypertension was attenuated in a genetic model of vascular smooth muscle Sirt1 overexpression (Castrejon‐Tellez et al., 2019; Fry et al., 2015; Gao et al., 2016). Likewise, smooth muscle cell‐specific Sirt1 overexpression and pharmacological activation of Sirt1 in a smooth muscle‐specific Sirt1 knockout model have been shown to protect against WD and high sucrose diet induced increases in arterial stiffness measured by PWV (Gao et al., 2016) and this vasoprotection was associated with reductions in vascular inflammation (Fry et al., 2016; Gao et al., 2016). In the present study, we used a global Sirt1 overexpression mouse model and examined aortic stiffness and structure to provide a more complete understanding of the impact of Sirt1 on WD associated increases in large artery stiffening. In agreement with the known benefits of Sirt1 overexpression, we observed that WD‐induced aortic stiffness was attenuated in Sirt1 transgenic mice compared with WT mice. As increases in aortic stiffness are an independent risk factor for CVD, our data suggests that interventions to increase Sirt1 activation may be efficacious to reduce arterial stiffening in obesity. This is in agreement with the previous studies, in which vascular smooth muscle specific (VSMC) and whole‐body Sirt1 overexpression protected against long term (8 months) diet‐induced aortic stiffening and further argues for the vasoprotective effects of Sirt1 (Fry et al., 2016).

The results of the present study expand upon this previous report by demonstrating that global overexpression of Sirt1 protects against short term (12 weeks) WD‐induced increases in large artery stiffness. Although we did not find any differential changes in collagen and elastin or aortic morphology in response to either Sirt1 overexpression or WD feeding, we observed transcriptional changes in matrix components and matrix metalloproteinases. Previous studies have also demonstrated that arterial stiffening ensues within 8–12 weeks of WD feeding in mice and that this was associated with vascular remodeling (Fry et al., 2016; Henson et al., 2014) and structural modifications in the arterial wall (Donato et al., 2013). In the present study, structural and morphological changes to the vessel wall cannot explain the protection against aortic stiffening afforded by Sirt1 overexpression. The extent to which these effects are facilitated by antioxidant and anti‐inflammatory activity as suggested by the smooth muscle specific pharmacological activation of Sirt1 (Fry et al., 2015, 2016) is unclear but requires further elucidation.

Although, we observed a decrease in collagen mRNA in WD fed Sirt^tg^ mice compared to WD fed WT mice (Figure 2a,b), we did not find that the Sirt1 overexpression impacted collagen content as expected from the observed changes in PWV. Furthermore, Movat's stained aortic sections demonstrated that proteoglycan was also unaffected by WD or Sirt1 overexpression (Figure 2). In addition, we found that WD led to higher expression of both MMP2 and MMP3 in WT mice, but this effect was absent in the Sirt^tg^ mice (Figure 2c,d). This finding is in agreement with previous studies (Medley et al., 2003; Yasmin et al., 2005) MMP 2 and 3 activity and gene modulation are associated with arterial stiffness. Taken together, this data suggests that post‐translational changes may underlie the lack of change in collagen protein expression observed despite altered mRNA, a possibility requiring further elucidation.

Furthermore, we find that Sirt1 overexpression‐mediated prevention of arterial stiffening in response to WD is associated with a maintenance of higher elastin mRNA expression compared to WT mice. However, as observed for collagen, this was not associated with a similar change in elastin quality when examined histologically, nor was the quality of elastin altered by WD or Sirt1 overexpression. Thus, although the maintenance of higher elastin mRNA expression in the Sirt1 transgenic mice in the face of WD is a potential contributor to the amelioration of WD induced arterial stiffening, it is not likely a primary factor. While it is unclear how the present study's findings of increased arterial stiffness and lower elastin expression in WD fed WT mice coincide, changes in vivo vascular tone is one possibility. While alterations in collagen and elastin may impact the mechanical properties of the vascular wall, impacting the material stiffness of the artery; but these changes may be masked in vivo by changes in arterial tone. Indeed, arterial stiffness is strongly affected by vascular tone, which is itself modified by endothelial function. In vivo, endothelial cell function can be modified by mechanostimulation, cell stretch, changes in calcium signaling, and by paracrine mediators including angiotensin II, oxidant stress, endothelin, and nitric oxide (Zieman et al., 2005). Although the role of altered vascular tone in the modulation of arterial stiffening by Sirt1 overexpression should be further explored, we and others have demonstrated that endothelial Sirt1 has a regulatory effect on vasodilation and vascular tone (Donato et al., 2011; Mattagajasingh et al., 2007; Wang et al., 2011) that may explain the decreases in WD induced arterial stiffening in the transgenic mice.

In the setting of WD‐induced obesity, elevated blood pressure has also been implicated in increased arterial stiffness via increases in pulse pressure and pulsatile aortic wall stress that can accelerate elastin degradation (Mitchell, 2014; Safar et al., 2006). Here, we did not observe an elevation in blood pressure after WD in either the WT or Sirt^tg^ mice (Figure 1). This finding is in agreement with other reports in the literature, in which 16 mo of 60% high fat feeding was found to be without effect on blood pressure in wildtype C57Bl/6 mice despite the mice demonstrating evidence of both vascular and cardiac dysfunction (Calligaris et al., 2013). In vivo changes in arterial tone or baroreceptor reflex activity may explain the disconnect between PWV and blood pressure observed, possibilities requiring further elucidation.

Mice fed a WD mimic human vascular and metabolic diseases and allow for the investigation of mechanisms underlying vascular consequences of obesity. Previously, we demonstrated arterial stiffness increases in the B6D2F1 mouse model in response to chronic WD feeding and aging (Henson et al., 2014). Although increased arterial stiffness is an independent risk factor for CVD, there is no specific treatment available to humans. Activation of Sirt1 may be a potential therapeutic target to treat elevations in arterial stiffness with either obesity or aging, as Sirt1 is abundantly expressed in vasculature and is involved in the maintenance of vascular homeostasis by providing protection against detrimental vascular tissue remodeling, atherosclerosis, and endothelial senescence (Bai et al., 2016; Chen, Yu, et al., 2020; Potente & Dimmeler, 2008; Wang et al., 2011) as well as in the regulation of vasodilation and vascular tone (Potente & Dimmeler, 2008; Tajbakhsh & Sokoya, 2012; Vaziri et al., 2001). Interestingly, Sirt1 is modulated in states of increased CVD risk such as aging and obesity. Indeed, Sirt1 expression decreases in endothelium with aging (Donato et al., 2011; Zu et al., 2010) and WD induces inflammation triggered cleavage of Sirt1 in adipose tissue which promotes metabolic dysfunction (Chalkiadaki & Guarente, 2012; Mariani et al., 2016; Sodhi et al., 2015). Although we cannot describe the direct mechanism in the present study, it is clear that Sirt1 overexpression results in a healthier vascular phenotype after WD. Our data demonstrate Sirt1 overexpression protects against WD associated glucose intolerance (Figure 5a) and this metabolic effect may be a contributor to the protection against arterial stiffening either through modulation of vascular tone via endothelial function or through changes to the matrix that may involve advanced glycated end products. Although these possibilities require direct examination, there is evidence in the literature to support these ideas. For example, previous studies have demonstrated that upregulation of Sirt1 improves aortic endothelial function in the setting of both hypercholesterolemia (Zhang et al., 2008) and high fat feeding (Fry et al., 2016) and Sirt1 has been reported to directly modulate endothelial function in small and large arteries through deacetylation and subsequent activation of eNOS, helping to maintain vascular homeostasis (Mattagajasingh et al., 2007). Likewise, aortic eNOS expression is higher in WD fed Sirt^tg^ mice compared to WD fed WT (Zhang et al., 2008). In addition to modulation of vascular tone, Sirt1 can modify the fibrotic effects of advanced glycated end products (AGEs) that are known to develop in the setting of metabolic dysfunction (Stirban et al., 2014). In addition to inducing oxidative stress and modulating transcription, AGEs can also act directly on the vascular wall contributing to an overproduction of matrix components (Stirban et al., 2014). However, Sirt1 overexpression has been shown to reduce AGE‐associated induction of fibronectin and transforming growth factor beta in the diabetic kidney (Huang et al., 2013; Kong et al., 2015). Taken together, these studies suggest that an endothelial‐driven reduction in arterial tone or inhibition of AGEs may underlie the protection against WD‐associated arterial stiffening in the Sirt1 overexpressing mice, a possibility requiring further elucidation. Nevertheless, in this present study, we demonstrated that overexpressing Sirt1 protects mice against arterial stiffening in response to WD, suggesting that Sirt1 activation may be a novel therapeutic target for treating obesity‐associated increases in arterial stiffness and deleterious arterial remodeling.

## FUTURE DIRECTION

Despite a lack of mechanistic insight into how Sirt1 modulated elastin, the findings presented demonstrate a specific effect on elastin, and not collagen, setting the stage for future studies aimed at elucidating the role of MMP 2 and 3 as mediator of the protective effects of Sirt1 overexpression on arterial stiffness.

## CONFLICT OF INTEREST

The authors have no conflicts of interest to disclose.

## ETHICS STATEMENT

All animal procedures conformed to the *Guide to the Care and Use of Laboratory Animals*: Eighth Edition (2011) and were approved by the Salt Lake City Veteran's Affairs Medical Center and University of Utah use Committees.

## AUTHORS CONTRIBUTION

Venkateswara R. Gogulamudi, Lisa A. Lesniewski, and Anthony J. Donato contributed to all aspects of the study; including the conception and design, data collection and analysis, and manuscript preparation. Daniel R. Machin, Grant D. Henson, Jisok Lim, Richard C. Bramwell, and Jessica R. Durrant contributed to the collection and analysis of data and revision of the manuscript.

## SUMMARY

The novel findings of this study reveals that Sirt1 overexpression prevents the aortic stiffening in response to WD feeding and suggest that this is associated with an increased elastin mRNA expression of the aorta. The findings suggest that Sirt1 plays an important protective role against WD induced large artery stiffening and may be a therapeutic target in the management of arterial stiffening in a metabolic disease population.
